# Carbamazepine-induced toxidermia: Case report and a literature review

**DOI:** 10.1192/j.eurpsy.2024.898

**Published:** 2024-08-27

**Authors:** R. Jbir, M. maalej, S. Omri, N. Charfi, R. Feki, I. Gassara, N. Smaoui, L. Zouari, J. Ben Thabet, M. Maalej

**Affiliations:** Psychiatry C department, Hedi chaker university hospital, Sfax, Tunisia

## Abstract

**Introduction:**

Carbamazepine is effectively used in treatment of bipolar disorder for its thymoregulatory virtues, but it can induce numerous side effects, including skin eruptions that can be severe sometimes.

**Objectives:**

To study the relationship between toxidermia and treatment with carbamazepine.

**Methods:**

We report the case of a patient who developed a toxidermia following the intake of carbamazepine.

**Results:**

Mr. AD, 19 years old, with medical history of diabetes, has been diagnosed with bipolar disorder since the age of 17. He was initially treated with risperidone with an irregular follow-up.

He was hospitalized in our department for a manic episode with psychotic features with agitation and refusal of treatment.

The patient was put on injectable treatment 15 mg/day of Haloperidol and 20 mg/day of diazepam.

After 5 days in hospital, we switched to the oral route, gradually increasing haloperidol doses to 30mg, reducing diazepam doses and introducing carbamazepine for thymoregulatory purposes.

Carbamazepine was progressively increased up to a dose of 800mg per day.

Fourteen days after the introduction of carbamazepine, the patient presented a generalized rash requiring the discontinuation of this medication. He was treated with an anti-histamine and local corticosteroids, on the advice of dermatologists.

In the days following discontinuation of carbamazepine, skin lesions regress and then disappear.

Biologically, we observed a rise in eosinophilic polynuclear cells to 580, followed by a gradual decrease after stopping the treatment.

A pharmacovigilance opinion was sought, concluding that carbamazepine was responsible for the toxidermia, given the delay in onset and the favorable evolution after discontinuation of the incriminating treatment. Moreover, this undesirable effect is well described in the literature.

Hence the contraindication to further use of carbamazepine in Mr. AD.

In addition, the patient was put on sodium valproate with good tolerance.

**Conclusions:**

Each prescribed drug must be considered as potentially capable of causing cutaneous reactions as an adverse effect. Both the prescriber and the patient must be made aware of this phenomenon. The attitude can be modulated on a case-by-case basis, after specialist advice, depending on the severity of the rash and the disease to be treated.

**Disclosure of Interest:**

None Declared

